# Early inflammatory events of mastitis—a pilot study with the isolated perfused bovine udder

**DOI:** 10.1186/s12917-021-03029-y

**Published:** 2021-11-19

**Authors:** Kathrin Susanne Brand, Viviane Filor, Wolfgang Bäumer

**Affiliations:** Institute of Pharmacology and Toxicology, Department of Veterinary Medicine, Freie Universität Berlin, Berlin, Germany

**Keywords:** Isolated perfused bovine udder, Mastitis, Lipopolysaccharide, Inflammation

## Abstract

**Background:**

Bovine mastitis is an important health and cost factor in the milk industry. To elucidate whether isolated perfused bovine udders can be used to study early inflammatory events of mastitis, 1 mg of lipopolysaccharide (LPS) was instilled into quarters of 10 isolated perfused bovine udders. Three hours and 6 h after LPS instillation, tissue samples were taken from the gland cistern and base of the udder, subsequently stored in RNAlater and processed for the determination of inflammation-dependent gene regulation by real-time RT-qPCR. Gene expression analysis was performed using delta-delta Ct method. To translate mRNA results to protein, IL-1ß and IL-6 were determined in tissue homogenate by ELISA.

**Results:**

The instillation of 1 mg LPS lead to an increased expression of pro-inflammatory cytokines and chemokines like TNF-α, CCL20, CXCL8 as well as of IL-1 ß, IL-6 and IL-10, lingual antimicrobial peptide (LAP) and S100A9. However, the degree of elevation differed slightly between gland cistern and udder base and markedly between 3 and 6 h after instillation, with a distinct increase in mediator expression after 6 h. IL-1β protein increased in a time-dependent manner, whereas IL-6 was unchanged within 6 h of LPS instillation.

**Conclusion:**

Compared to in vivo studies with instillation of LPS into udders of living cows, a similar inflammation-dependent gene regulation profile can be mimicked in the isolated perfused bovine udder, indicating a supplementation of animal experiments.

**Graphical abstract:**

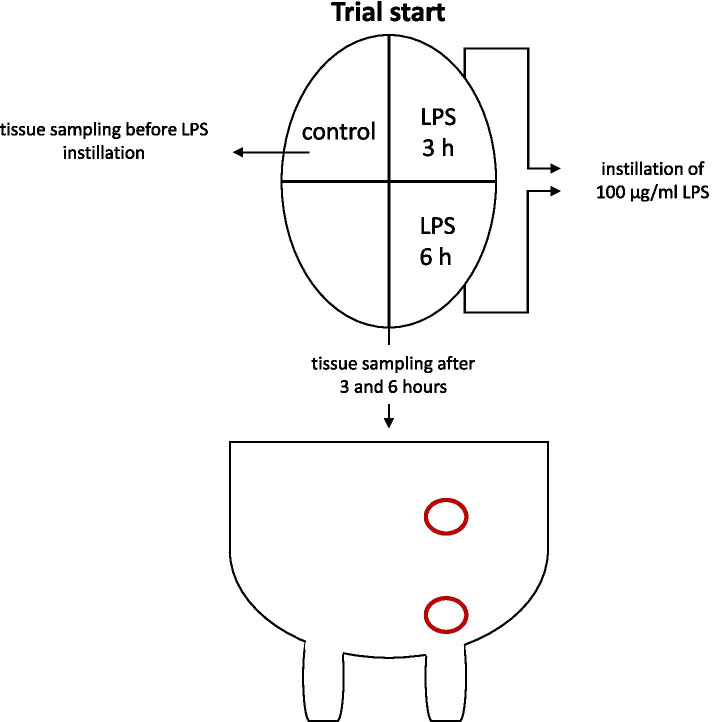

**Supplementary Information:**

The online version contains supplementary material available at 10.1186/s12917-021-03029-y.

## Background

Mastitis, defined as an inflammatory process of udder tissue mainly due to bacterial infection, is a major health and cost burden in dairy cattle worldwide [1, 2]. Reduced milk production due to mastitis and its associated treatment is a substantial financial burden in the dairy industry. Intramammary infections with *Escherichia coli, Streptococcus uberis* and *Staphylococcus aureus* in particular differ in their immunological response and can lead to severe and or long lasting damage of the tissue, which is associated with high production losses [1, 3]. Clinical signs of mastitis include swelling due to painful edema, acute inflammation and, following chronic development, fibrosis of udder tissue [2].

Although recent gene expression profiles of tissue from mastitis cows have been performed, early events of infection and inflammation in particular are still not fully understood [4, 5].

Thus, we wanted to perform a proof of principle study to find out whether the isolated perfused bovine udder may be a suitable tool to study early inflammatory events in mammary tissue.

As a first stimulus, lipopolysaccharide (LPS) as part of gram-negative cell walls from e.g. *E. coli* was used. The signal transduction of LPS is well characterized and includes an interaction with lipopolysaccharide binding protein, CD14, toll like receptor (TLR) 4 and Myeloid Differentiation factor 2 (MD-2) [6]. This in turn leads to the regulation of many genes involved in acute inflammation and infection. There have been formerin vitro and in vivo experiments data for LPS and/or E. coli stimulation response.In vitro in primary isolates from bovine mammary epithelial cells have been stimulated and in vivo, there are published LPS instillation trials in cows [7, 8]. Thus, we intended to assess the value of isolated perfused bovine udders as an *ex-vivo* model ranging in complexity between monolayer cell cultures and a living organism. The selection of possible regulated genes (cytokines TNFα, IL-1β, IL-6, IL-10, chemokines CCL20, CXCL8 and antimicrobial effector molecules LAP and S100A9) were adapted from a recent *in-vivo* study that also focussed on early inflammatory events in mastitis [4].

## Results

The regulation of the inflammatory genes is always given in comparison to the control. The control is a sample before LPS stimulation (time 0) to avoid possible interactions between the udder quarters after LPS stimulation.

### Regulation of inflammatory genes 6 h after the instillation of 100 µg LPS

In pilot experiments, different concentrations of LPS were tested (udders from three different cows; *N* = 3). As 100 µg LPS were used in in vivo experiments [5], we started with 100 µg/quarter in 10 ml sterile NaCl 0.9%.

**Fig. 1:**
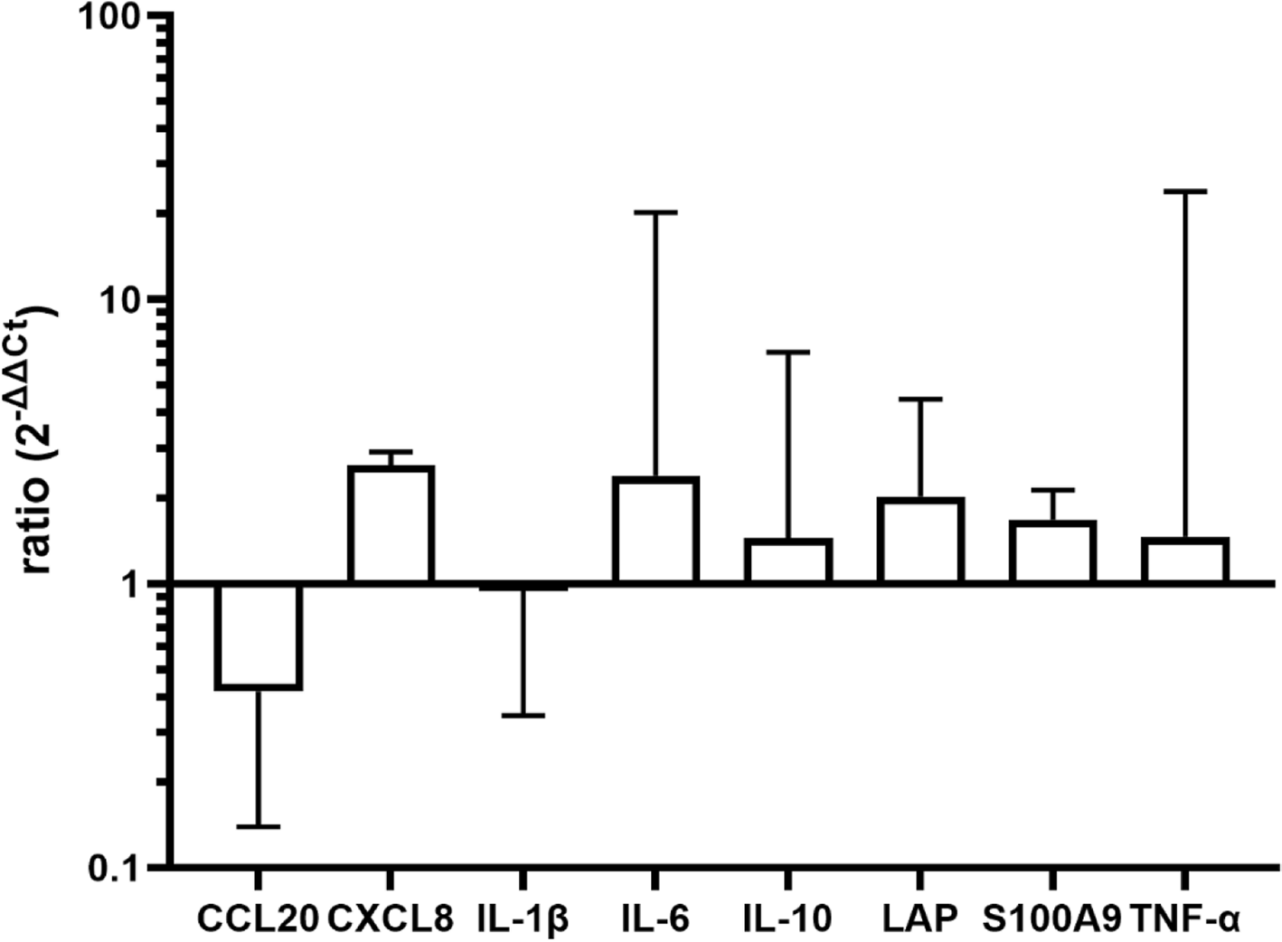
Expression of CCL20, CXCL, IL-1, IL-6, IL-10, LAP S100A9 and TNF-α (ddCT/ GAPDH) 6 h after instillation of 100 µg LPS in 10 ml PBS in the gland cistern. *N* = 3, dotted line represents control level

Although there was a slight increase noticeable 6 h after the instillation (Fig. 1, results for udder base are shown here), the overall response was only moderate. It was thus decided to increase the concentration of LPS to 1000 µg/quarter for the main experiment.

### Regulation of inflammatory genes 3 h after the instillation of 1000 µg LPS

**Fig. 2:**
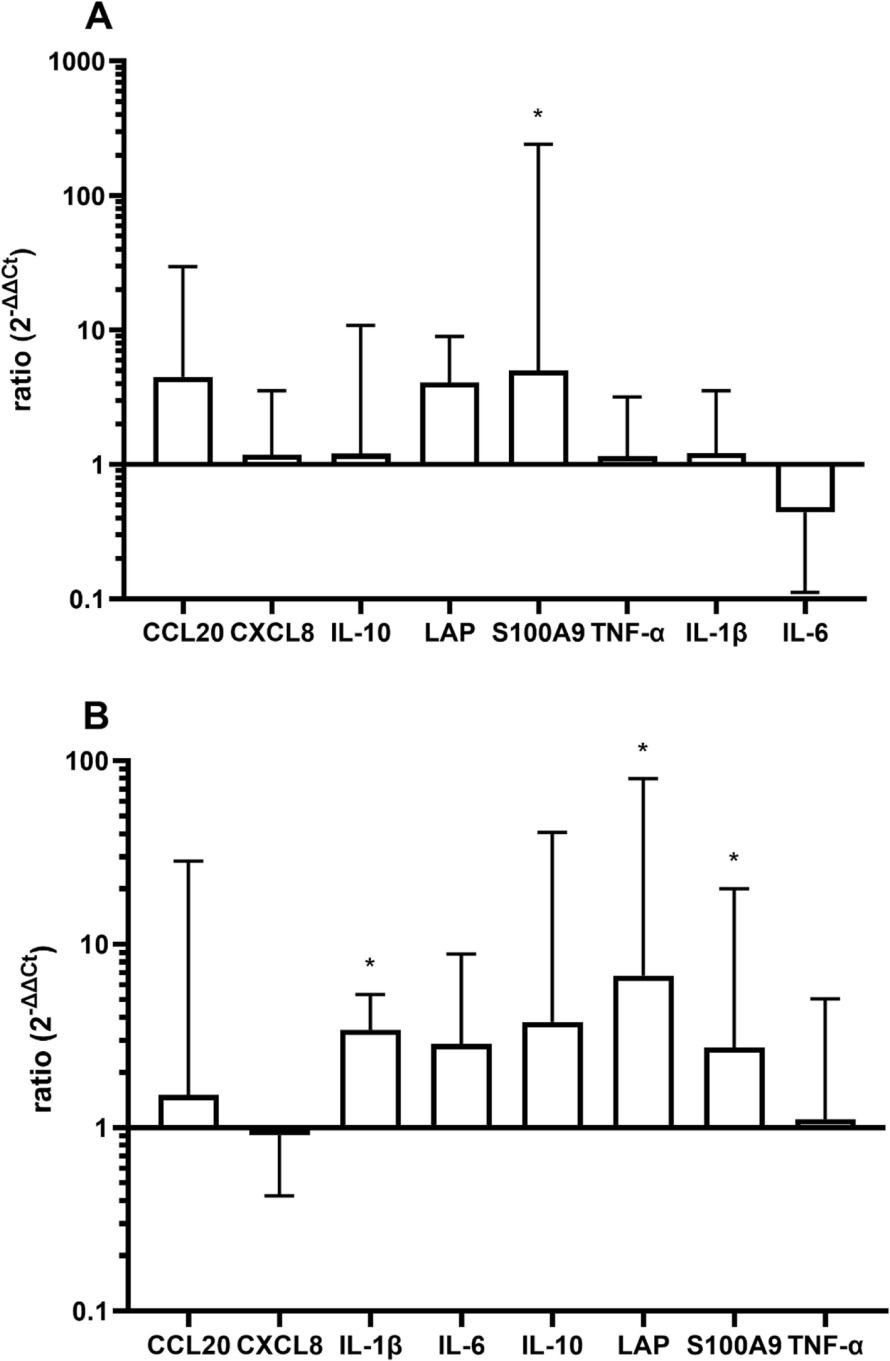
Expression of CCL20, CXCL, IL-1, IL-6, IL-10, LAP S100A9 and TNF-α (ddCT/ GAPDH) 3 h after instillation of 1000 µg LPS in 10 ml PBS in the gland cistern (**A**) and udder base (**B**). *N* = 10, dotted line represents control level.*p < 0.05 compared to control

Figure 2 shows the regulation of inflammatory genes 3 h after stimulation with LPS on udders from 10 different cows (*N* = 10, *p < 0.05 compared to control). There was generally an increase of transcription of pro-inflammatory mediators (particularly CCL20, LAP and S100A9). The overall response was marginally stronger at the udder basis (Fig. 2B) where three effector molecules (LAP and S100A9) were significantly increased, whereas in the gland cistern, (Fig. 2A), only S100A9 was significantly increased 3 h after LPS instillation.

### Regulation of inflammatory genes 6 h after the instillation of LPS

**Fig. 3:**
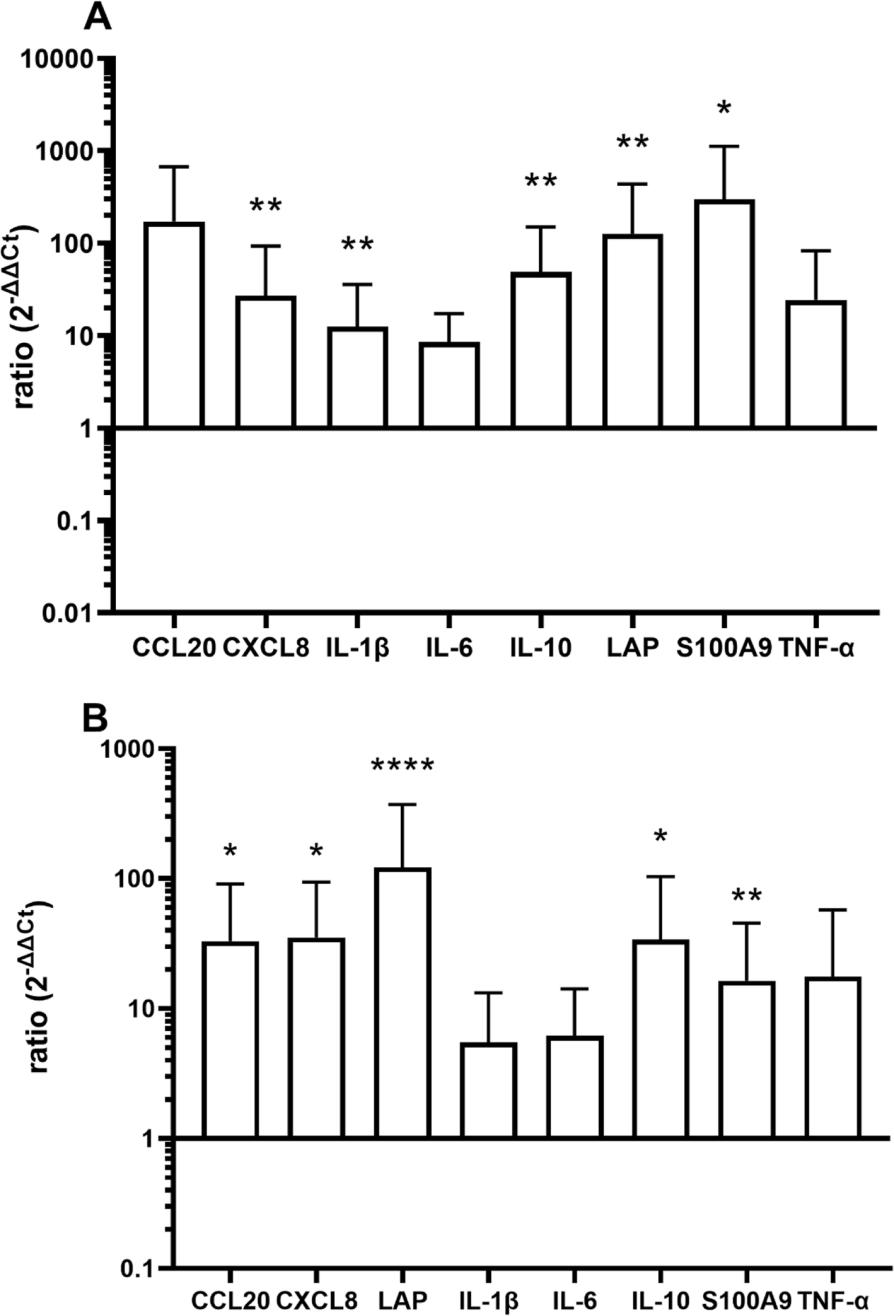
Expression of CCL20, CXCL, IL-1β, IL-6, IL-10, LAP S100A9 and TNF-α (ddCT/ GAPDH) 6 h after instillation of 1000 µg LPS in 10 ml PBS in the gland cistern (**A**) and udder base (**B**). *N* = 10, dotted line represents control level.*p < 0.05, **p<0.01, ****p<0.0001 compared to control

The increase of transcription was more pronounced 6 h after LPS instillation. This became significant for CXCL8, β IL-1β, IL-10, and antimicrobial effector molecules LAP and S100A9 in the gland cistern (Fig. 3A) and for CXCL8, CCL20, IL-10, LAP and S100A9 in the udder base (Fig. 3B). Udders from 10 different cows (*N* = 10) were also included in the data analysis in these experiments.

### IL-1β, IL-6 and PGE2 concentration in tissue homogenate 3 and 6 h after the LPS instillation

**Fig. 4:**
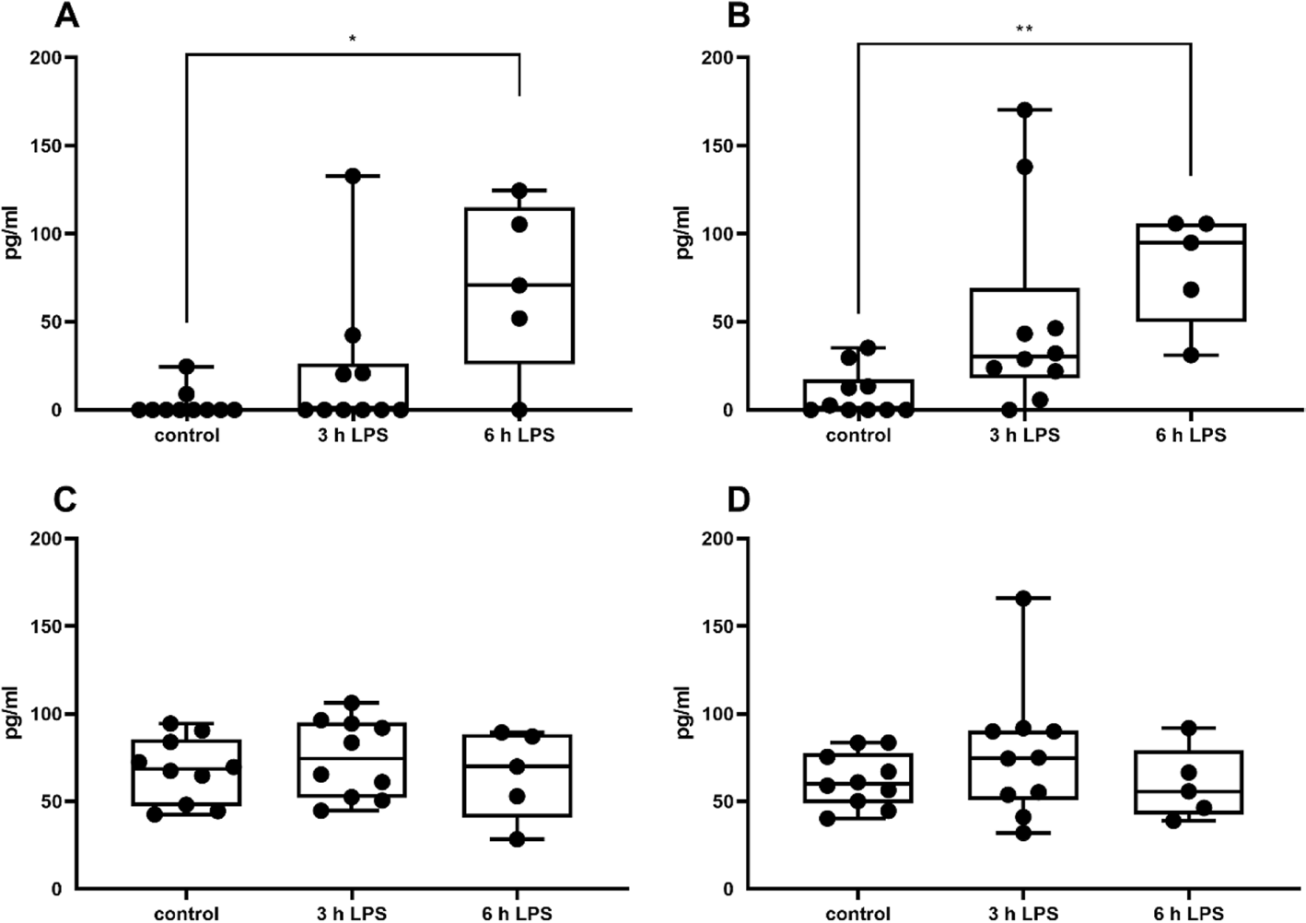
Concentration of IL1-β in tissue of gland cistern (**A**) and udder base (**B**) and of IL-6 in tissue of gland cistern (**C**) and udder base (**D**) 3 h and 6 h after instillation of 1000 µg LPS into the udder quarters. *N* = 5 (6 h) to 10 (control and 3 h), *p < 0.05, ** *p* < 0.01

**Fig. 5:**
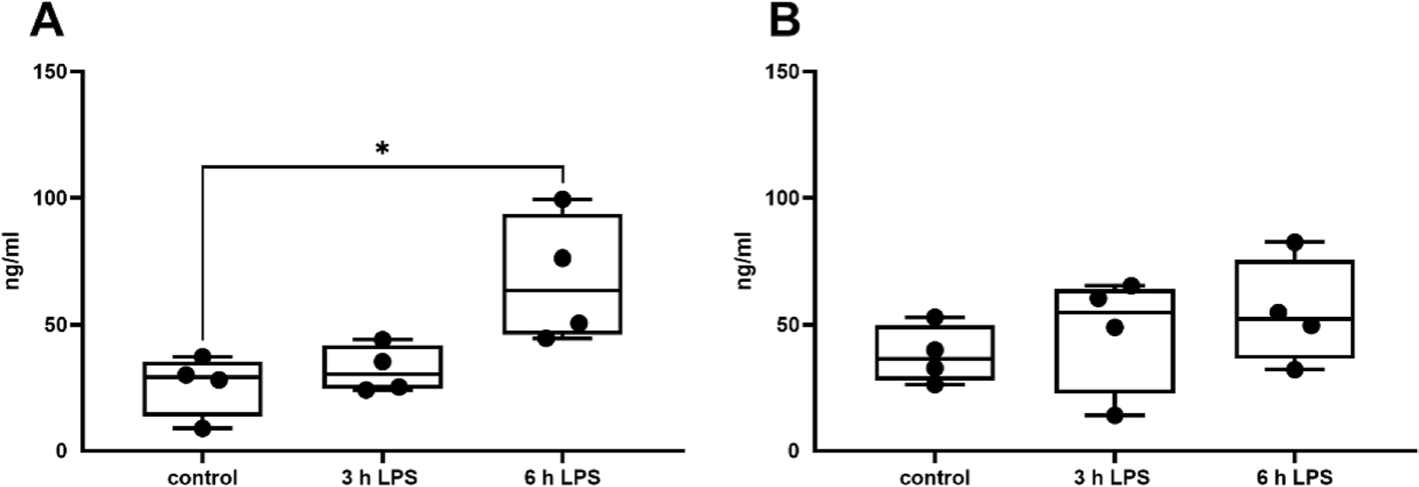
Concentration of PGE_2_ in tissue of gland cistern (**A**) and udder base (**B**) 3 h and 6 h after instillation of 1000 µg LPS into the udder quarters. *N* = 4, data are given as median and all the samples analysed are represented as dots. *p < 0.05

The concentration of bovine IL-1β increased in a time dependent manner in both the gland cistern and the udder base (Fig. 4A/B) and became significantly increased 6 h after the LPS instillation. When PGE_2_ concentration was determined, a significant increase was observed after 6 h in the glandular cistern, but not in the udder base (Fig. 5A/B). Although a measurable concentration of bovine IL-6 was observed in control samples, there was no further increase of IL-6 after 3 or 6 h of incubation with LPS (Fig. 4C/D). Data for IL-1ß and IL-6 measurements from 10 cows for 3-h values (*N* = 10) and 5 cows for 6-h values (*N* = 5) were included. The PGE_2_ examinations were obtained from 4 cows (*N* = 4).

### Altered gene regulation in “mastitis” quarters

During the study, five udder quarters from four different cows were identified with a highly elevated cell count. These were not used for LPS experiments, but tissue samples were taken directly at the beginning of the perfusion, in order to evaluate whether these “mastitis” quarters show an altered expression of the selected cytokines.

**Fig. 6:**
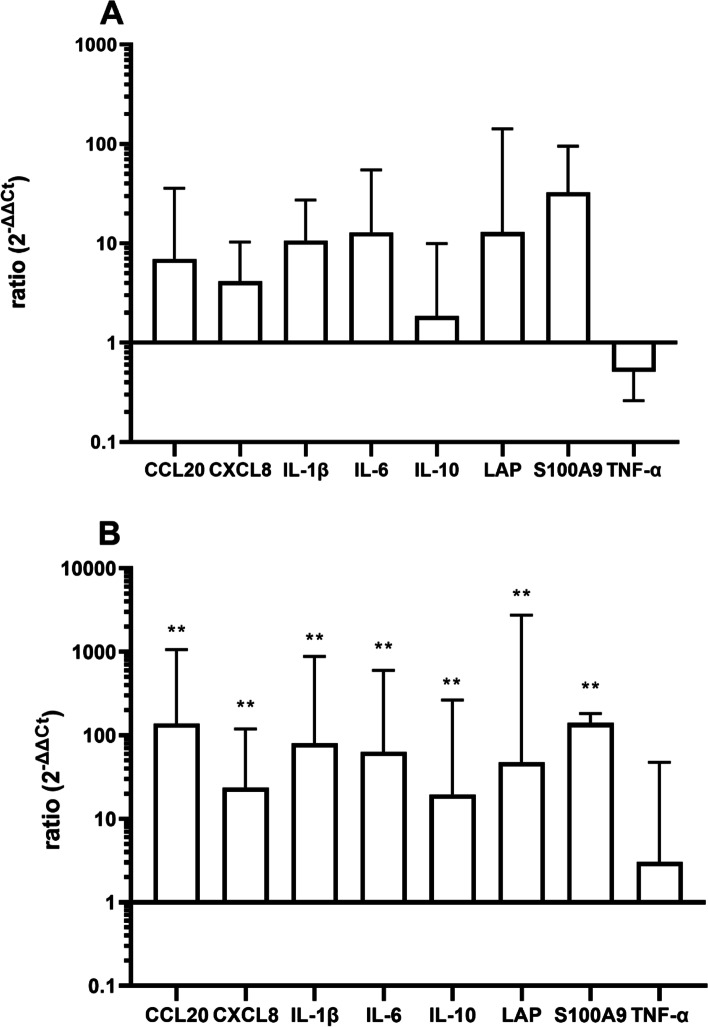
Expression of CCL20, CXCL, IL-1, IL-6, IL-10, LAP S100A9 and TNF-α (ddCT/ GAPDH) in udder quarters with high somatic cell count at the beginning of the perfusion in the gland cistern (**A**) and udder base (**B**). *N* = 5, dotted line represents control level. *p < 0.05, **p < 0.01 compared to control

Interestingly, the pattern of alterations were similar to that seen after the LPS instillation. However, the alterations in the udder base were much more pronounced compared to the altered expression at the gland cistern (Fig. 6A/B). It should be noted that we cannot say with certainty which pathogen triggered the increased cell count. We want to take this fact into account and investigate it in future experiments.

## Discussion

The aim of the present study was to gain first proof of principle data in isolated perfused bovine udders as a potentially feasible model for early inflammatory events during mastitis. The hypothesis of the current study was that inflammatory responses in isolated perfused bovine udders range between simple two-dimensional cell cultures and complex in vivoexperiments [4, 7]. The hypothesis of the current study was that investigations of bovine mastitis using the model of the isolated perfused bovine udder may bridge a gap between simple two-dimensional cell cultures and complex in vivo experiments.

It was stressed in former studies that the response of mammalian epithelial cells (MECs) to LPS or inactivated bacteria do not mimic all responses observed in the infected udder in vivo*.* One striking difference is the lack of IL-10 mRNA upregulation in MECs, whereas a strong induction was described in udders [9]. Even in the SV40 immortalized and frequently used bovine mammary alveolar cell line MAC-T, IL-10 was not detectable [10]. Thus, the increase of IL-10 observed in the isolated bovine udder indicates that the response is closer to the in vivo situation compared to simple 2D cells cultures [4]. In addition, the up-regulation of S100A9 is also very moderate in MEC compared to in vivoin udders. A first hint that S100A9 is significantly secreted by the cells of the udder was given by the results of Lind et al. (2015) [11]. After LPS stimulation, an up-regulation of S100A9 was observed using teat explants. Taken together, the response in the isolated udder might be closer to the in vivosituation. On the other hand, LAP, CCL20 and CXCL8 did belong to the top 20 upregulated genes in the pbMECs [9], as well. Although recent publications indicate that TNF-α is associated with an LPS-induced inflammatory response, Günther et al., (2009) [9] could not identify TNF-α as one of the top 20 up-regulated genes in their pbMEC studies. However, studies are contradictory: TNF-α detection was possible in experiments with the LPS-stimulated cell line MAC-T [12–14]. Lind et al. (2015) [11] showed a TNF-α baseline and, in addition, a LPS-dependent TNF-α expression in primary cells using a teat explant model. In our results, only a slight increase in TNF-α was observed, however, results obtained by Rabot et al. (2007) [15] show similar findings. Based on literature, these results seem surprising and may be attributed to variable individual immune responses.

When first experiments in udders were accomplished, study results of ex vivo infection experiments in isolated perfused udders have been published that support the feasibility to use udders as a meaningful model [16, 17]. Nevertheless, we believe that the current data are still valuable, as the LPS stimulation allows a direct comparison with former in vitro and in vivo studies and helps to further evaluate the values of isolated perfused bovine udders in mastitis research.

In contrast to in vivo infection with *E. coli*, where an early response (1 and 3 h after infection) was observed in the glandular cistern in particular, in the present LPS stimulation, there are only marginal differences between responses in udder base and gland cistern after 3 h. This might be explainable by the different cause of stimulation, instilled *E. coli* still grow during infection (5 × 10^6^ CFU at the beginning of infection to roughly 5 × 10^8^ CFU three hours after infection) [4]. This initial proliferation might mainly occur in the cistern, whereas the instillation of LPS in sterile NaCl 0.9% might lead to a faster more even distribution up to the udder base.

Limitations of the study.

The great variability of response in isolated udders has to be seen as a limitation. Interestingly, there were no “low” and “high” responding udders in general (i.e. in one udder the CCL20 response was high, but the CXCL8 response low) but rather a variability within and between the udders. Thus, a sampling size of around 10 udders seems reasonable to gain robust responses.

Protein data show that mRNA and protein correlate, at least as shown here for IL-1β. Interestingly, we saw at least the tendency of an IL-6 increase, particularly after 6 h (however, this did not become significant). This underscores that mRNA and protein data do not always have to correlate. Nevertheless, we investigated the presence of IL-6 and IL-1ß by ELISA in one “mastitis” quarter. IL-6 was increased sevenfold in the gland cistern and a threefold increase was seen in the udder base compared to a healthy udder quarter. In contrast, IL-1ß could not be detected (data not shown). Prostaglandins (e.g. PGE_2_) play an important role in acute inflammatory processes. Therefore, we subsequently decided to investigate PGE_2_ by using an ELISA kit. The data obtained from 4 udders show a significant increase of the PGE_2_ concentration in the udder cistern 6 h after LPS treatment. This result demonstrates that the processes of acute inflammation can be mimicked in the model of the isolated perfused udder and motivate us to consider this fact in more detail in future studies.

It is intended to use isolated udders for possible prevention experiments (e.g. testing of new anti-infective or anti-inflammatory strategies like biofilm preventer or bacteriophages, as well). Although there is a definite time limitation of maximal 8 h of perfusion, these inflammatory response results support the usefulness of isolated perfused udders to study and modify early inflammatory processes of mastitis. For long-term examinations, the model of precision-cut bovine udder slices (PCBUS) can be used [18]. In this model, cells remain to their original location-specific composition and also resemble the in vivo situation. Additionally, more trails can be performed with tissue of one udder, since the generation of approximately 200 PCBUS from one udder quarter is possible. Filor et al. (2021) [18] demonstrated a stimulability of PCBUS by LPS through the differential release of immune mediators by ELISA. The combined use of both models can achieve complementary results in the field of bovine mastitis for future studies.

## Conclusion

These first proof of principle data indicate that early inflammatory responses of mastitis can be monitored in isolated perfused bovine udders and that this model can be used to supplement in vivo mastitis experiments.

## Material and methods

### Isolated perfused bovine udder

The udders of slaughtered cows (German Holstein Friesian) were immediately examined at the slaughterhouses by inspection and palpation for udder health. Since the udders came from different slaughterhouses in the area, we do not have preliminary reports on the animals. The perfusion of bovine udders was performed as described in the previous studies [19, 20]. In brief, immediately after healthy cows had been slaughtered, the organs were collected. Only udders that had no visible skin lesions or pathological changes of the milk and glandular tissue (macroscopic and palpatoric examination) were utilized. Directly after slaughter, milk samples were collected from the udder so they were available for cell counting with a Neubauer chamber in the laboratory. For somatic cell count a threshold of 100.000 cells/ml was set [21]. Furthermore, a so-called California Mastitis Test (CMT) was also performed directly on site, as this is another indicator of udder health. Both were performed before perfusion with the tyrode. To avoid clot formation, a first perfusion was performed at the slaughterhouse with roughly 2 L of tyrode solution after the cannulation of the right and left external pudendal arteries. For transit 300 ml heparinized Tyrode solution was instilled in each udder half. After transport to the institute, the mamma complexes were perfused via the external pudendal arteries with carbogen saturated tyrode solution possible by means of a peristaltic pump (Masterflex 7518 10; Cole Parmer Instr., Chicago, U.S.A.) with flux rate 100 to 120 ml per minute. The perfusion fluid was tempered at 38.5 °C. The perfusion started within 45 to 60 min after slaughtering. For the determination of glucose consumption, a venous drainage via the *vena epigastrica cranialis superficialis* was performed. For this purpose, samples from venous perfusate were collected and analysed at time 0 and then every 2 h until the end of the experiment. The viability of the perfused udder was demonstrated by a nearly unchanged glucose consumption.

### Challenge with lipopolysaccharide

To exclude subclinical mastitis, cell count in milk was measured in each quarter with a Neubauer chamber. Only udder quarters < 100.000 somatic cells/ml were taken for instillation experiments.

Depending on the number of healthy udder quarters, one to three quarters were instilled with a concentration of 100 µg/ml LPS (O55:B5, Sigma-Aldrich, Steinheim, Germany) in 10 ml sterile NaCl 0.9%. Different quarters were used for 3 h experiments and 6 h experiments. In total, udder quarters from 14 different cows were included in the analysis. Samples were taken from the respective areas using sterile forceps and a scalpel blade. A skin incision was made and the glandular tissue was retrieved from inside the organ using forceps and cut off with the scalpel. To exclude any influence of LPS on the untreated quarters, it was decided to take control samples from glandular cistern and udder base from one quarter directly before the instillation of LPS. In pilot experiments it was verified that the expression of the studied genes did not alter during 6 h of perfusion (data not shown).

Udder tissue samples were taken from the glandular cistern and udder base 3 h and 6 h after LPS instillation. To minimize leakage after the incision of udders, the incisions for control samples and 3 h after the instillation were closed by a simple suture technique.

### Samples from quarters with high cell count

Five udder quarters from four different cows had high or very high somatic cell counts (between 2 × 10^5^ and 5 × 10^6^ cells/ml) and thus were not used for LPS instillation trials. However, tissue samples were taken directly at the beginning of the perfusion from these quarters to evaluate whether these “mastitis” quarters show an altered expression of the selected inflammation-associated genes.

### Isolation of mRNA and RT-qPCR technique

Excised udder tissue (ca. 50 mg) was stored in RNAlater (Thermo Fisher Scientific GmbH, Dreieich, Germany) at 4 °C overnight and then frozen at -80 °C until further analysis. For mRNA extraction, samples were homogenised by means of a T 25 Ultra-Turrax (Ika, Staufen, Germany) on ice, followed by a further homogenization with a sterile syringe and cannula. Lysis and mRNA isolation (including DNA digestion) was performed with RNeasy Mini Kit as well as RNase-Free DNase Kit (Qiagen, Hilden, Germany) according to manufacturer`s protocol. Transcription to cDNA was carried out with QuantiTect Rev. Transcription Kit (Qiagen) and cDNA was stored at -20 °C. The quality and purity of RNA was evaluated at 260/280 nm wave-length (~ 1.8) and 260/230 nm wave-length (~ 2.0 – 2.2).

For RT-qPCR Maxima, SYBR Green/Fluorescein RT-qPCR-Mastermix was used with a cDNA concentration of 200 ng/sample. Selected primers and sources are presented in Suppl. Table 1. For RT-qPCR 40 cycles were conducted, after an initial denaturation at 95 °C for 10 min as follows: denaturation at 95 °C for 15 s. followed by annealing and extension at 60 °C for 60 s. At completion, melting curves were monitored for quality assurance and also, gels were run to confirm the correct amplification size.

An analysis of experiments was performed according to the delta/delta CT method. As house-keeping genes Glyceraldehyde-3-phosphate dehydrogenase (GAPDH) and 18 s rRNA were compared. GAPDH led to very robust results and thus was used for all further analysis as house-keeping gene.

### Protein extraction and determination of IL-1β, IL-6 and PGE_2_ by ELISA

About 400 mg of glandular tissue sample were taken from udder base and glandular cistern before, 3 h and 6 h after LPS instillation and instantly frozen at -80 °C. As it was decided to take samples for protein analysis after the experiments already started, for the 6-h LPS instillation, only samples from 5 udders were available, whereas 10 udders were available for the 3-h values. Additionally, prostaglandin E_2_ (PGE_2_) concentrations could be investigated in 4 udders as only tissue from 4 udders were left, when this additional determination was performed.

Samples were thawed in 1 ml T-PER™ Tissue Protein Extraction Reagent + Protease Inhibitor (Thermo Fisher Scientific) and homogenised with the T 25 Ultra-Turrax (Ika) on ice.

After centrifugation (3000 g, 10 min, 4 °C) protein content was measured (BCA Protein Assay Kit, Cell Signaling Technology Europe B.V., Frankfurt am Main, Germany) in supernatant and samples stored at -20 °C. Concentrations of bovine IL-1β (Thermo Fisher Scientific GmbH), bovine IL-6 (Duo Set, R&D Systems, Minneapolis, USA) and PGE_2_ (Prostaglandin E2 Express ELISA Kit Cayman Chemical Company, Ann Arbor, USA) were determined by ELISA according to the manufacturer`s protocol.

### Statistical analysis

The results were analysed to determine whether there was a significant difference in mRNA and protein content between untreated and LPS treated udder quarters (gland cistern and udder base) 3 h and 6 h after the instillation. Three hours vs. control and 6 h vs. control were compared with the non-parametric test (Mann Whitney U-test) for significant difference. If more than two groups have been compared, a Kruskal Wallis-Test was performed, followed by multicomparison test (Dunn`s test). *P* < 0.05 was set as significance level. The statistical evaluation was performed with GraphPad Prism (Version 9.0.2, GraphPad Software, Inc.).

## Supplementary Information

**Additional file 1: Supplemental Table 1.** Oligonucleotide primers for RT-qPCR.

## Data Availability

The datasets used and/or analysed during the current study are available from the corresponding author on reasonable request.

## Abbreviations

GAPDH: Glyceraldehyde-3-phosphate dehydrogenase
IL: Interleukin
LAP: Lingual antimicrobial peptide
LPS: Lipopolysaccharide
MEC: Mammalian epithelial cell
NaCl: Sodium chloride
PCBUS: Precision-cut bovine udder slices
PGE_2_: Prostaglandin E_2_
TLR: Toll like receptor
