# Supplementary material for: Early inflammatory events of mastitis—a pilot study with the isolated perfused bovine udder
Source: BMC Vet Res. 2021 Nov 19;17:356. doi: 10.1186/s12917-021-03029-y (PMC8603483; doi:10.1186/s12917-021-03029-y)
Supplement: Supplementary file 1 — Additional file 1: Supplemental Table 1. Oligonucleotide primers for RT-qPCR. [file 12917_2021_3029_MOESM1_ESM.docx]

Supplemental Table 1: Oligonucleotide primers for RT-qPCR.

| **Target** | **Primer sequences^1^ (5’ → 3’)** | **Product size (bp)** | **GenBank no.** |
| --- | --- | --- | --- |
| **GAPDH** | ACATCGTCGCCATCAATGACC | 207 | NM_001034034.2 (novel design) |
|  | ACTCCACCACATACTCAGCACC |  |  |
| **CCL20** | GACTGCTGTCTCCGATATACA | 71 | NM_174263.2  (LIND et al., 2015) |
|  | GCCAGCTGCTGTGTGAAGC |  |  |
| **CXCL8** | CCTCTTGTTCAATATGACTTCCA | 189 | NM_173925.2 (Macías Luaces, L. 2019) |
|  | GGCCCACTCTCAATAACTCTC |  |  |
| **IL-1β** | AAAAATCCCTGGTGCTGGCT | 88 | [NM_174093.1  (novel design)](https://www.ncbi.nlm.nih.gov/nuccore/NM_174088.1) |
|  | ATGCAGAACACCACTTCTCGG |  |  |
| **IL-6** | AAGCGCATGGTCGACAAAAT | 164 | NM_173923.2  (novel design) |
|  | AAGCAAATCGCCTGATTGAACC |  |  |
| **IL-10** | TGACTTTAAGGGTTACCTGGGTT | 131 | [NM_174088.1 (Macías Luaces, L. 2019)](https://www.ncbi.nlm.nih.gov/nuccore/NM_174088.1) |
|  | GCTTCTCCCCCAGTGAGTTC |  |  |
| **LAP** | AGGCTCCATCACCTGCTCCTT | 183 | NM_203435.4  (PETZL et al., 2016) |
|  | CCTGCAGCATTTTACTTGGGCT |  |  |
| **S100A9** | CTCAAACAGAAGGCGGGAAA | 225 | NM_001046328.2 (PETZL et al., 2016) |
|  | TTGTGTCCAGATCCTCCAT |  |  |
| **TNF-α** | CTTCTGCCTGCTGCACTTCG | 156 | NM_173966.3  (YANG et al., 2008) |
|  | GAGTTGATGTCGGCTACAACG |  |  |
| ^1^Upper line: forward primer, lower line: reverse primer. | | | |
